# Nickel‐chelatase activity of SirB variants mimicking the His arrangement in the naturally occurring nickel‐chelatase CfbA

**DOI:** 10.1002/2211-5463.13849

**Published:** 2024-06-24

**Authors:** Yuuma Oyamada, Shoko Ogawa, Takashi Fujishiro

**Affiliations:** ^1^ Department of Biochemistry and Molecular Biology, Graduate School of Science and Engineering Saitama University Saitama Japan

**Keywords:** chelatase, mutagenesis, nickel, porphyrin, protein–ligand docking

## Abstract

Metal–tetrapyrrole cofactors are involved in multiple cellular functions, and chelatases are key enzymes for the biosynthesis of these cofactors. CfbA is an ancestral, homodimeric‐type class II chelatase which is able to use not only Ni^2+^ as a physiological metal substrate, but also Co^2+^ as a nonphysiological substrate with higher activity than for Ni^2+^. The Ni/Co‐chelatase function found in CfbA is also observed in SirB, a descendant, monomeric‐type class II chelatase. This is despite the distinct active site structure of CfbA and SirB; specifically, CfbA shows a unique four His residue arrangement, unlike other monomeric class II chelatases such as SirB. Herein, we studied the Ni‐chelatase activity of SirB variants R134H, L200H, and R134H/L200H, the latter of which mimics the His alignment of CfbA. Our results showed that the SirB R134H variant exhibited the highest Ni‐chelatase activity among the SirB enzymes, which in turn suggests that the position of His134 could be more important for the Ni‐chelatase activity than that of His200. The SirB R134H/L200H variant showed lower activity than R134H, despite the four His residues found in SirB R134H/L200H. CD spectroscopy showed secondary structure denaturation and a slight difficulty in Ni‐binding of SirB R134H/L200H, which may be related to its lower activity. Finally, a docking simulation suggested that the His134 of the SirB R134H variant could function as a base catalyst for the Ni‐chelatase reaction in a class II chelatase architecture.

## Graphical abstract

CfbA is a class II chelatase that utilizes Ni^2+^unlike other class II chelatases, such as SirB. The structural uniqueness of CfbA is due to the four His residues at its active site. We studied SirB variants mimicking this His arrangement and showed that a His‐mediated base catalysis is key for the Ni‐chelatase activity in class II chelatases.

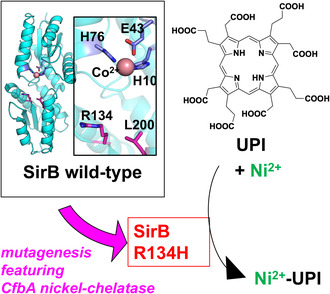

**Abbreviations**
CD: circular dichroism
DTT: dithiothreitol
IPTG: isopropyl‐β‐D‐thiogalactopyranoside
LB: Luria‐Bertani
SHC: sirohydrochlorin
UPI: uroporphyrin I

Chelatases are key enzymes for the biosynthesis of metal–tetrapyrrole cofactors and can catalyze the insertion of a divalent metal ion into a substrate macrocyclic tetrapyrrole [1]. There are three distinct classes of chelatases: classes I, II, and III [2]. Class II chelatases comprise the largest group among all the classes and exhibit various structures and functions [3, 4]. Most class II chelatases are monomers and exhibit a large active site pocket with tetrapyrrole‐ and metal‐binding sites. The structures of these binding sites are suitably arranged for their substrate selectivities. For example, mammalian FECH ferrochelatase uses protoporphyrin IX (PPIX) and Fe^2+^ [5, 6, 7, 8, 9, 10, 11, 12, 13, 14]. SirB is also a ferrochelatase in siroheme biosynthesis; however, its tetrapyrrole substrate is sirohydrochlorin (SHC) rather than PPIX [2, 15, 16, 17, 18]. Some cobalt‐chelatases can also utilize SHC with Co^2+^ in cobalamin biosynthesis [3, 15, 19, 20, 21, 22].

More recently, CfbA has been identified as the naturally occurring nickel‐chelatase catalyzing Ni^2+^ insertion into SHC in coenzyme F430 biosynthesis [23, 24]. CfbA has several unique structural features compared with other class II chelatases [2, 3, 4, 25, 26]. Uniquely, CfbA exhibits a homodimeric architecture that can be superposed onto the overall structure of other monomeric class II chelatases. Within the active site of CfbA, two metal‐binding sites are symmetrically located. In *Methanocaldococcus jannaschii* CfbA (*Mj* CfbA) [26], one subunit provides two conserved His9 and His75 residues for one metal‐binding site, while the other subunit also has another pair of His9 and His75. Overall, four His residues are arranged within the active site cavity. In addition, the SHC‐binding area exists between two pairs of His9 and His75. Under low Ni^2+^ concentrations, one metal‐binding site is sufficient for catalysis, in which the other pair of His residues may function as a base catalyst for SHC deprotonation, after binding of SHC and Ni^2+^ to the active site (Fig. 1). The coordination geometry for Ni^2+^ of CfbA has been studied by X‐ray crystallography (Fig. 2). Three residues, His9, His75, and Glu42 are likely to be positioned in a facial manner when the Ni‐coordination is assumed as a 6‐coordination.

**Fig. 1:**
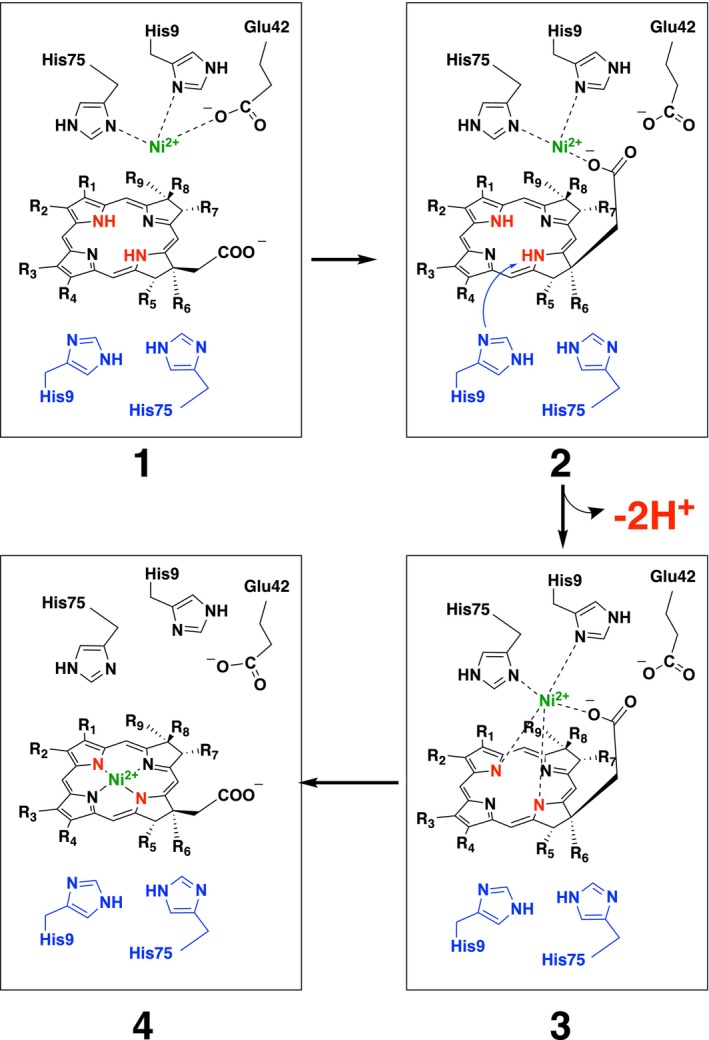
Proposed mechanism of nickel‐chelatase reaction by CfbA with sirohydrochlroin (SHC) and Ni^2+^ involved in His‐mediate base catalysis. First, SHC is bound to the active site, followed by Ni^2+^‐binding to a pair of His9 and His75 with the support of Glu42. Then, an acetate of SHC is bound to Ni^2+^ via ligand exchange with Glu42, as captured by X‐ray crystallography [26]. This ligand exchange may contribute to SHC ring distortion, which is favorable for His‐mediated base catalysis to abstract protons from SHC, although the necessity of either His9 or His75, or both His9 and His75, has not been clarified so far. Finally, Ni^2+^ is inserted into SHC, yielding Ni‐sirohydrochlorin. The SHC's substituents R_n_ (*n* = 1–9) are as follows: R_1_ = R_4_ = R_8_ = CH_2_COO^−^, R_2_ = R_3_ = R_5_ = R_7_ = CH_2_CH_2_COO^−^, R_6_ = R_9_ = CH_3_.

**Fig. 2:**
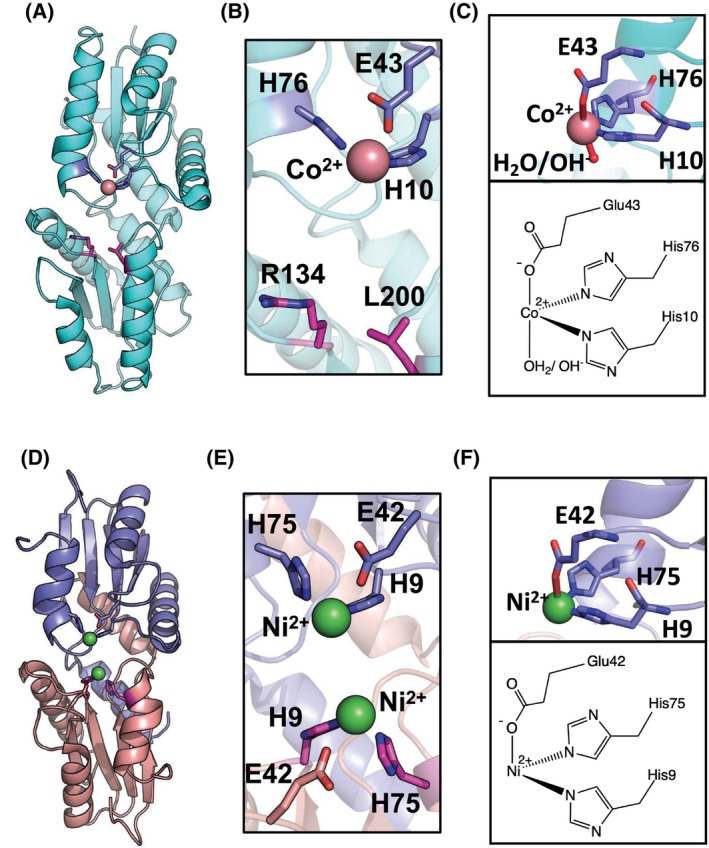
Structures of SirB and CfbA. (A) Overall and (B) active site structures of SirB in complex with Co^**2**+^ (PDB ID: 5ZT7) [18]. (C) View for the geometry of the Co^**2**+^‐binding site and its chemical structure. (D) Overall and (E) active site structures of CfbA in complex with Ni^**2**+^ (PDB ID: 6M27) [26]. (F) Geometry of the Co^**2**+^‐binding site and its chemical structure. Residues for metal binding in the N‐terminal domain of SirB and the corresponding residues of CfbA are colored slate blue. Mutated residues of the C‐terminal domain of SirB and their corresponding residues of CfbA are colored magenta. Notably, CfbA exhibits the homodimeric architecture colored in slate blue and pink for one and the other of the monomers. The overall homodimeric architecture of CfbA resembles the SirB monomeric architecture. Cobalt and nickel ions are shown in pink and green spheres, respectively. The CfbA structure contains two Ni^**2**+^ but only one Ni^**2**+^ bound to the blue‐colored residue is sufficient for the nickel‐chelatase reaction. The binding of the other Ni^**2**+^ is attributed to the high Ni^**2**+^ concentration in X‐ray crystallographic analysis [26]. It should be noted that Co^**2**+^‐ and Ni^**2**+^‐coordinations in these SirB and CfbA structures, especially the presence of H_2_O or OH^−^ ligands, are not completely resolved, although one H_2_O or OH^−^ is visible in the case of SirB. However, by considering the facial geometry of three amino acid ligands (two His and one Glu) in SirB and CfbA as well as the preference of Co^**2**+^‐ and Ni^**2**+^‐coordinations in water, there could be 4, 5, or 6‐coordination.

Considering the His‐mediated base catalysis and previously determined X‐ray crystal structures of CfbA with SHC and/or Ni^2+^, a catalytic mechanism of nickel‐insertion to SHC by CfbA has been proposed (Fig. 1) [26]: First, the SHC molecule is bound to the active site, followed by binding of Ni^2+^ to one pair of His9 and His75 with a supporting ligand of Glu42 (**1** of Fig. 1). Then, an acetate group of SHC is bound to Ni^2+^ via ligand‐exchange instead of Glu42 (**2** of Fig. 1), which is captured by X‐ray crystal structure of a reaction intermediate of CfbA [26]. This ligand exchange may be able to make SHC distorted, which makes NH moieties face on the His9 and/or His75, which are different from Ni^2+^‐bound His9/His75. Afterwards, His‐mediated base catalysis by His9 and/or His75, which is a focus of this study, is contributing to deprotonating of SHC (**2** of Fig. 1). After the deprotonation of SHC, Ni^2+^ can be inserted into the central pyrrole‐nitrogens of SHC (**3** of Fig. 1), yielding Ni‐sirohydrochlorin as a product (**4** of Fig. 1).

It is known that CfbA can utilize not only Ni^2+^, but also Co^2+^ as a metal substrate [2, 26, 27]. The activity for Co^2+^ of CfbA is much higher than Ni^2+^. The Ni/Co‐chelatase function is also known in some monomeric‐types of class II chelatases, such as SirB [15]. However, the active site structures of CfbA and SirB are rather distinct (Fig. 2), e.g. the numbers of conserved His residues serving as metal‐binding ligands and/or possible base catalysts to abstract protons from their tetrapyrrole substrate. Currently, it is interesting how the arrangement of the four His residues in CfbA contributes to nickel‐chelatase activity. The uniqueness of the His arrangement of CfbA can be recognized via comparison to monomeric‐type of class II chelatases, such as SirB. For example, SirB has only two His (Fig. 2), which resembles a pair of two His residues for Ni‐binding in CfbA (Figs. S1 and S2), although other parts of the residues of SirB are different from the corresponding ones of CfbA (Fig. 2). To consider the relationship between the His residue arrangement and nickel‐chelatase activity, we herein studied monomeric SirB variants mimicking CfbA's His arrangement. We prepared three SirB variants, R134H, L200H, and R134/L200H variants, and characterized them using Ni^2+^ and uroporphyrin I (UPI), an SHC analog used for facile nickel‐chelatase assay [18]. Circular dichroism (CD) spectroscopy was performed to gain clues to consider the possible structure–function relationship of the SirB variants, especially the R134H/L200H variant showing lower activity than expected. Furthermore, docking simulation to make models of UPI‐bound SirB WT and the variants was performed for addressing a possible role of His residues as a base catalyst, which may be related to increased Ni‐chelatase activity of the SirB R134H variant than SirB WT.

## Materials and Methods

### Materials

UPI and myoglobin were purchased from Sigma‐Aldrich (St. Louis, MO, USA). Isopropyl‐β‐D‐thiogalactopyranoside (IPTG) was purchased from BLD Pharmatech (Shanghai, China). Dithiothreitol (DTT), imidazole, and chloramphenicol were purchased from FUJIFILM‐Wako Pure Chemical (Tokyo, Japan). All other chemicals were purchased from Nacalai Tesque (Kyoto, Japan). All DNA oligo primers (Table S1) were purchased from Eurofins Genomics Japan (Tokyo, Japan). Ferritin, aldolase, conalbumin, and ovalbumin were purchased from Cytiva (Tokyo, Japan).

### Site‐directed mutagenesis

For the site‐directed mutagenesis of SirB, the pACYC‐*sirA‐His‐sirB‐sirC* plasmid [18], which is used for the expression of *Bacillus subtilis* SirB wildtype (WT) with an N‐terminal His_6_‐tag, was used as a template for inverse PCR with mutagenic primers. The amplified PCR products were digested with DpnI and self‐ligated with a 2× ligation convenience kit (Nippongene, Tokyo, Japan) and T4 polykinase (Nippongene). The ligation mixture was used to transform *Escherichia coli* DH5α cells. Transformed *E. coli* DH5α colonies were selected in Luria–Bertani (LB) medium supplemented with 25 μg/mL chloramphenicol and then cultivated at 37 °C for 12 h. The plasmid was extracted from cultivated *E. coli* DH5α cells and used for DNA sequencing of *sirB* to check for the mutation of interest. To construct the expression systems of SirB R134H, L200H, and R134H/L200H variants, three plasmids (pACYC‐*sirA‐His‐sirB R134H‐sirC*, pACYC‐*sirA‐His‐sirB L200H‐sirC*, and pACYC‐*sirA‐His‐sirB R134H/L200H‐sirC*) were used to transform *E. coli* C41(DE3).

### Expression and purification of SirB WT and three variants

SirB WT and the variants were expressed and purified as previously described [18]. Transformed *E. coli* C41(DE3) cells harboring either of the expression plasmids for WT or the variants were cultivated in 6 L of LB medium supplemented with 25 μg/mL chloramphenicol at 37 °C for 4 h. When the OD_600_ value reached 0.4–0.6, IPTG was added to the culture at a final concentration of 1 mM. The culture was incubated at 20 °C for 20 h to express the SirB variants. Subsequently, *E. coli* cells were harvested via centrifugation at 9000 ***g*** for 20 min at 4 °C. The harvested *E. coli* cells were frozen in liquid nitrogen and stored at −80 °C.

SirB WT and the variants were purified either on ice or at 4 °C. *E. coli* cells expressing either SirB WT or the variants were disrupted via sonication. The sonicated cells were then centrifuged at 20 000 ***g*** for 40 min at 4 °C. The supernatant was then loaded onto a HisTrap FF crude column (Cytiva) equilibrated with buffer A (50 mM Tris–HCl, pH 7.8, 500 mM KCl, and 1 mM DTT). After washing the column with buffer A, SirB was eluted with buffer B (50 mM Tris–HCl, pH 7.8, 500 mM KCl, 1 mM DTT, and 250 mM imidazole). The pooled SirB fractions were concentrated using Amicon Ultra‐15 (Merck‐Millipore, Burlington, MA, USA). The concentrated SirB was loaded onto a HiPrep 16/60 Sephacryl S‐200 HR column equilibrated with buffer C (50 mM Tris–HCl, pH 7.8, 150 mM NaCl, and 1 mM DTT). SirB fractions were pooled and concentrated before further use. The purity of SirB was evaluated via SDS‐PAGE (Fig. S3).

### Gel filtration analysis for determining the molecular weight of SirB

Oligomeric states of SirB WT and three variants in solution were performed by gel filtration chromatography using a Superdex™ S200 Increase 10/300 GL column installed onto Akta Go (Cytiva) at 4 °C with a flow rate of 0.3 mL/min. The column was equilibrated with buffer D (50 mM Tris–HCl buffer pH 7.8, 150 mM NaCl, 1 mM tris(2‐carboxyethyl)phosphine) For the analysis of either of SirB WT or variant samples, temperature, 100 μL of SirB was injected. Absorption at 280 nm was monitored to gain chromatograms and values of elution volumes of SirB samples. Calibration for molecular mass calculation was created by gel filtration analysis of molecular weight protein markers: ferritin (Mw = 440 000), aldolase (Mw = 158 000), conalbumin (Mw = 75 000), ovalbumin (Mw = 43 000), and myoglobin (Mw = 17 000). Using the calibration, the molecular weight of SirB WT and three variants were calculated (Fig. S4).

### Activity assay

For the nickel‐chelatase activity assay, the reaction mixture was prepared as follows: 5 μM SirB WT or either of the variants, 50 μM UPI, and 200 μM NiCl_2_ in a reaction buffer (50 mM Tris–HCl, pH 8.0, 150 mM NaCl) in a total volume of 10 μL of the reaction mixture, and their reaction was performed in the dark. The UV–visible spectra of the reaction mixtures were measured on the Implen UV–visible C40 spectrophotometer equipped with a submicroliter cell with a 1 mm cell path (Implen, Munich, Germany). The UV–visible spectrum of each reaction mixture was scanned from 400 to 700 nm. Furthermore, the changes in the absorbance at 552 nm, attributed to the product nickel–uroporphyrin I (Ni–UPI), were plotted over time. The absorbance at 552 nm after a 24‐h reaction was used to calculate the relative nickel‐chelatase activity. The ratio of the change in the absorbance at 552 nm (Δ*A*_552_) of an SirB variant/WT was calculated. Thus, the relative activity for WT was 1.0. Notably, for the SirB R134H variant, curve‐fitting using a pseudo‐first‐order kinetic equation was possible, whereas it was not possible for the other datasets for WT, L200H, and R134H/L200H. The pseudo‐first‐order rate constant (*k*) for the SirB R134H variant with UPI and Ni^2+^ was calculated via nonlinear root mean square curve‐fitting to the plots with Igor Pro 8.0 (WaveMetrics, Lake Oswego, OR, USA). The reactions for each plot were repeated at least three times (*n* = 3) and standard deviations (SD) were represented as error bars at each point. UV–visible spectrum of Ni^2+^ solution (10 mM NiCl_2_ in 50 mM Tris–HCl buffer, pH 7.8) was recorded on an Implen C40 spectrophotometer with a 1 cm‐path quartz cuvette to check the peaks derived from Ni^2+^ alone.

### Docking simulation

Docking of UPI to SirB WT and the variants was performed using AutoDock Vina [28] on UCSF Chimera (http://www.cgl.ucsf.edu/chimera). The model structures of the SirB R134H and L200H variants were prepared by substitution of the amino acids of interest (i.e. Arg134 and Leu200) with His in the X‐ray crystal structure of SirB WT (PDB ID: 5ZT7) [18] in open‐source PyMOL (Schrödinger, New York, NY, USA). When preparing the SirB models, cobalt ions and water molecules were removed from the SirB WT and variant models. The UPI ligand model was prepared using PRODRG [29] via the energy‐minimizing process for optimizing its conformation. Each of the SirB WT or variant and UPI models was set as a rigid receptor and ligand for Autodock Vina, respectively. The residues Arg134, or His134, and Leu200, or His200, were set as flexible residues. The UPI model was also set to have flexible conformations for its substituents, i.e. acetates and propionates. The docking parameters were similar to those used in a previous study [18]. The initial coordinate for UPI was set to (x, y, z) = (39.703, −10.618, 12.017). The size of the docking grid box was 72.0 Å × 44.0 Å × 58.0 Å. The other docking parameters were used as the default. After the docking simulation, the highest‐ranked structure based on the computed binding energy was selected as a favorable docking model. All protein figures are represented using PyMOL.

### CD spectroscopy

CD spectroscopy on SirB WT and variants were performed using the J‐1500 CD spectrophotometer (JASCO, Tokyo, Japan) with a quartz cuvette with a 1‐cm cell path. In all the measurements, the temperature was maintained at 20 °C. The number of residues in SirB was 273, which was used to calculate the mean residue molar ellipticity, [*θ*] = (recorded degrees/cell path × protein molar concentration × residue number).

For measurement of CD spectra derived from secondary structure regions of SirB WT and variants, SirB WT and the three SirB variants in 50 mM Na‐phosphate buffer (pH 7.8) were prepared, to result in 3 mL of sample volume for each case. The protein concentrations of SirB WT and the R134H, L200H, and R134H/L200H variants were 1.28, 0.93, 1.34, and 1.97 μM, respectively.

For measurement of the near UV–visible range of the CD spectra of SirB WT and variants with NiCl_2_, a quartz cell with a volume of 1 mL was used. NiCl_2_ was titrated to prepare mixtures of 10, 20, 30, 40, or 60 μM of NiCl_2_ and SirB WT or variants. For the near UV–visible range of CD spectroscopy, protein concentrations of SirB WT and the R134H, L200H, and R134H/L200H variants were 12, 17, 19, and 18 μM, respectively.

## Results

### SirB variants mimic the His residues in the CfbA active site

To design SirB variants mimicking CfbA, we first compared the active sites of *Bacillus subtilis* SirB (*Bs* SirB) and *Mj* CfbA [18, 26] (Fig. 2). The metal‐binding site of *Bs* SirB contains His10, Glu43, and His76, which are at the equivalent positions to His9, Glu42, and His75 of *Mj* CfbA. However, *Mj* CfbA has a second set of His9, Glu42, and His75. Instead, Arg134 and Leu200 of *Bs* SirB are equivalently located at the second set of His9 and His75 of *Mj* CfbA. Furthermore, the Glu of CfbA (e.g. Glu42 of *Mj* CfbA) was not conserved, as observed in the amino acid sequence alignment of CfbA enzymes [26]. For example, *Archaeoglobus fulgidus* CfbA (*Af* CfbA), which is also called *Af* CbiX^S^ [3, 27], has Ala instead of the Glu. In other words, the pair of two His residues are strictly conserved among CfbA enzymes, whereas the Glu is not. Therefore, we focused on His arrangement rather than the Glu, with Arg134 and Leu200 as the target sites for mutagenesis. This resulted in the construction of three *Bs* SirB variants: R134H, L200H, and R134H/L200H. Each of these *Bs* SirB variants was purified with good purity (Fig. S3) and eluted as a monomer (Fig. S4) in the gel filtration analysis, as in the case of *Bs* SirB WT.

### Nickel‐chelatase activity of SirB variants

**Fig. 3:**
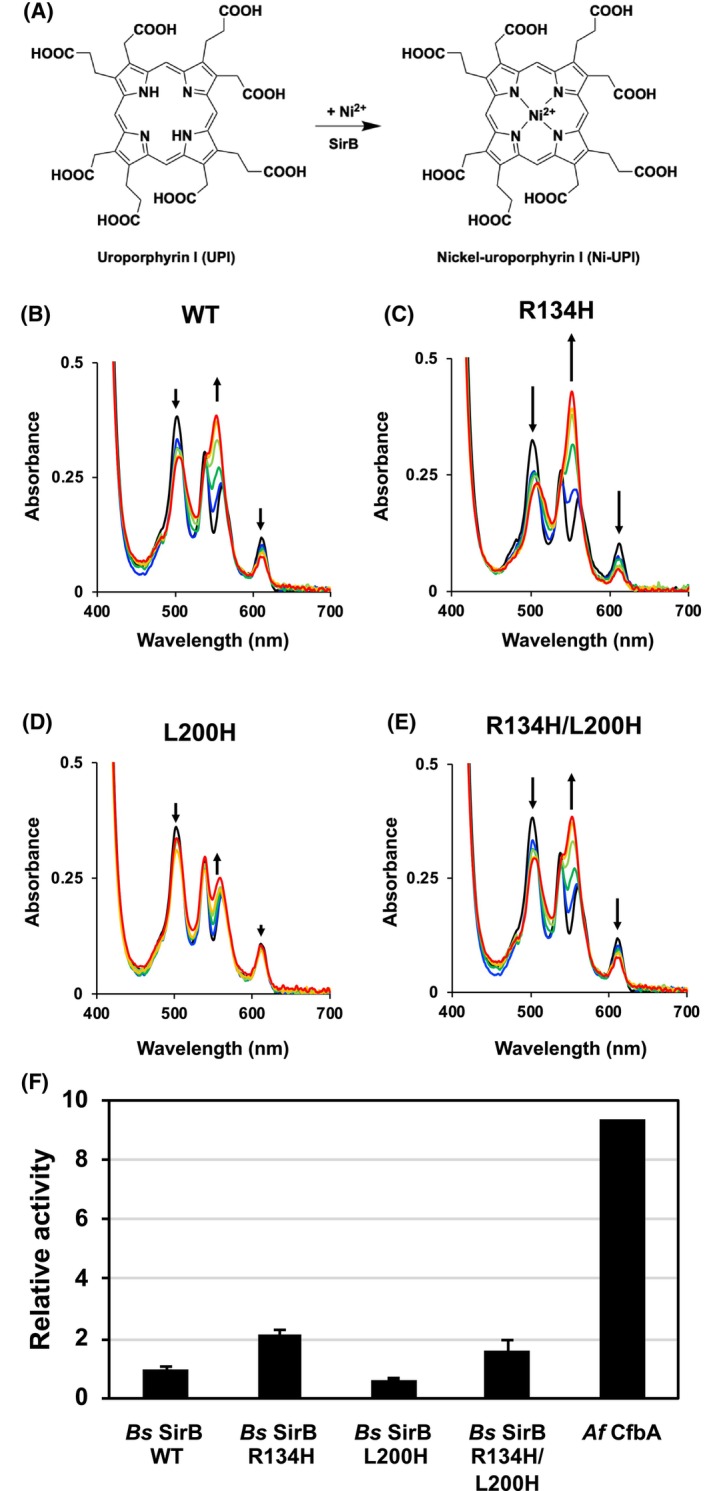
(A) Scheme for UPI–nickel–chelatase reaction. (B–E) Changes of UV–visible spectra in reaction mixtures of UPI, Ni^2+^ with (B) SirB WT, (C) SirB R134H variant, (D) SirB L200H variant, and (E) SirB R134H/L200H variant. Black lines of UV–visible spectra indicate the spectra just after initiating the reaction (0 h). The changes of the absorption peaks are indicated by black arrows. The time‐course changes of the UV–visible spectra are colored in blue (6 h), green (12 h), light green (18 h), orange (22 h), and red (24 h). The values of the UV–visible peak absorbance at 552 nm, derived from the product Ni–UPI, were used to calculate the relative activity. The relative activity of the WT was set to 1.0. Error bars indicate the standard deviations. Reactions for each set of conditions were performed three times. (F) Comparison of the relative Ni‐chelatase activity for UPI. The relative nickel‐chelatase activity of *Af* CfbA (*Af* CbiX^S^) for UPI was also calculated by using its activity value, which was reported previously [30].

The nickel‐chelatase activity of the three *Bs* SirB variants was analyzed by inserting nickel into UPI, a commercially available SHC analog (Fig. 3A) [18]. *Bs* SirB WT exhibited slight nickel‐chelatase activity for UPI with Ni^2+^. This was similar to the findings of a study on the nickel‐chelatase activity of SHC with Ni^2+^ [15]. Next, we studied the nickel‐chelatase activities of the SirB variants with UPI. Among them, *Bs* SirB R134H exhibited the highest nickel‐chelatase activity, followed by *Bs* SirB R134H/L200H (Fig. 3). The activities of these two SirB variants were higher than those of WT. In contrast, the nickel‐chelatase activity of the SirB L200H variant was lower than that of WT. Furthermore, no reaction for Ni–UPI formation was observed without SirB (Fig. S5A). The observed UV–visible spectra of the mixture of SirB, Ni^2+^ and UPI were different from of Ni^2+^ alone (Fig. S5B). These data also supported that the observed nickel‐chelatase reactions were catalyzed by SirB WT and the variants.

Thereafter, the activity of the SirB R134H variant was analyzed using the pseudo‐first‐order kinetic equation via nonlinear root mean square curve‐fitting (Fig. S6); as a result, the apparent kinetic constant (*k*) was 6.6 × 10^−4^ ± 2.6 × 10^−4^ min^−1^. The *k* value of the *Bs* SirB R134H variant in the Ni–UPI formation reaction was 4.4‐fold lower than that of *Af* CfbA (*k* = 2.9 × 10^−3^ min^−1^) (Fig. 3F) [30]. In contrast, the activities of the other SirB variants and WT could not be analyzed in the same manner as those of SirB R134H, owing to their extremely low activities.

### Partial denaturation of *bs* SirB R134H/L200H

**Fig. 4:**
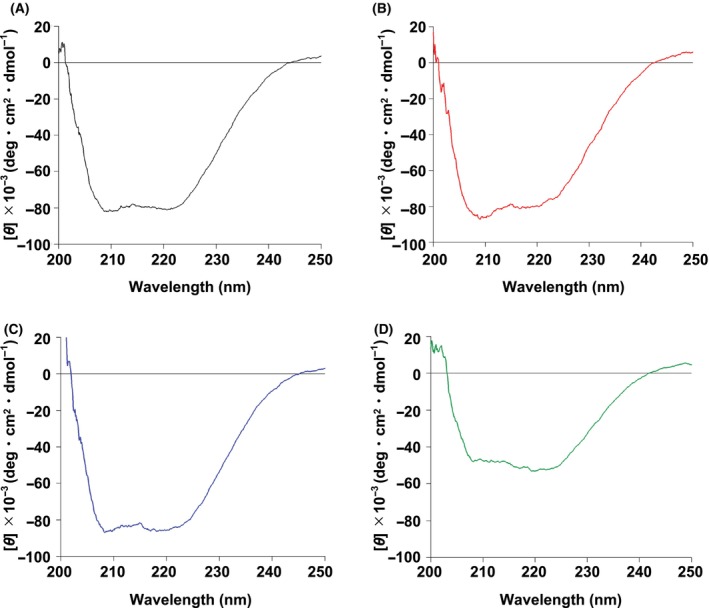
CD spectra of SirB in a range of 200–250 nm. (A) WT and the (B) R134H, (C) L200H, and (D) R134H/L200H variants.

Next, *Bs* SirB WT and the three variants were characterized using CD spectroscopy (Fig. 4). The WT, R134H, and L200H variants exhibited almost identical CD spectra by comparison of their double minimum spectra derived from α‐helices in the range of 200–250 nm. In contrast, the *Bs* SirB R134H/L200H variant exhibited decreased α‐helix peaks. The content of the secondary structures of the *Bs* SirB R134H/L200H variant was ~50%–60% lower than those of *Bs* SirB WT and the other variants. Therefore, CD spectroscopy of the three SirB variants revealed that the structure of the R134H/L200H variant may be different from that of the other variants.

To gain further insights into the protein structure–function relationship, we performed near UV–visible range of CD spectroscopy of SirB WT and variants with Ni^2+^ (Fig. S7). The CD spectral peak at 279 nm increased upon the addition of Ni^2+^ to SirB WT or variant. This peak at 279 nm could be considered as the ligand‐to‐metal charge transfer (LMCT) in a similar manner to CooJ, a Ni‐chaperon using His residues for binding Ni^2+^ [31]. Thus, the CD spectral changes at 279 nm could indicate that Ni‐binding occurred in all the cases of SirB. Although the quantitative analysis of the binding constant of Ni^2+^ to SirB samples could fail due to poor intensity of this spectral changes at this region, it was clear that the R134H/L200H variant could show a spectral change only at a higher concentration of Ni^2+^ (30 μM) compared with the other SirB enzymes in the presence of 20 μM of Ni^2+^. This could be interpreted as the lower‐affinity of R134H/L200H variant to Ni^2+^. If this feature of Ni‐binding of R134H/L200H variant may have been caused by a partial denaturation around the Ni‐binding sites, including not only H134H/H200, but also His10/His76, the lowered nickel‐binding affinity could be understood. In such a case, a largely denatured signature found in CD spectroscopy on 200–240 nm of R134H/L200H variant could also be understood: The effect of mutation on R134H/L200H may have been made the region of His10/His76 as well.

### Docking simulation to the SirB variant models with UPI

**Fig. 5:**
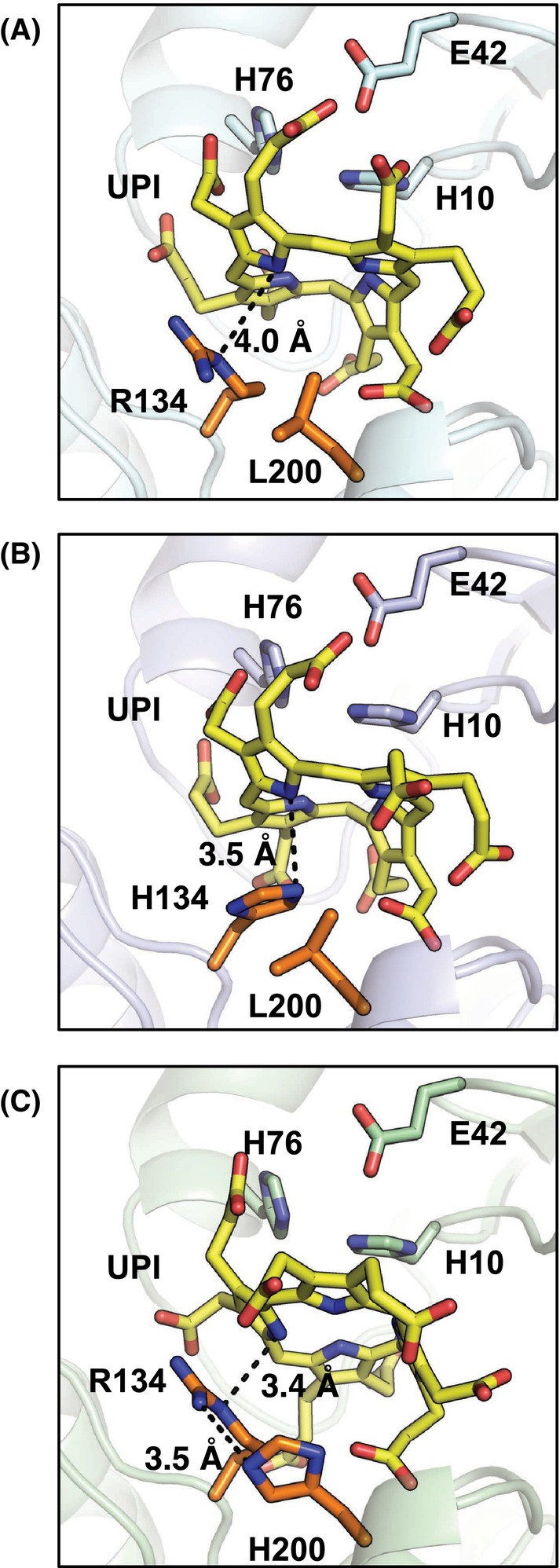
Active site structures of the docking models of SirB with UPI. (A) SirB WT, (B) SirB R134H, and (C) SirB L200H. Black dashed lines indicate the distances. Yellow stick models indicate UPI. Dashed lines indicate possible polar interactions between UPI and Arg134, His134, Leu200, or His200, which are represented as orange‐colored stick models.

The structural models of UPI‐bound *Bs* SirB variants were computed to understand why the R134H mutation is involved in enhancing nickel‐chelatase activity (Fig. 5). In the UPI‐docked model of *Bs* SirB R134H, the NH groups of UPI were positioned adjacent to the metal‐binding site and His134. The position of His134 was suitable to function as a base catalyst at neutral or weakly basic pH, resulting in deprotonation of the NH groups of UPI toward enhanced activity. In the UPI‐docked model of *Bs* SirB WT, the ε‐NH moiety of Arg134 interacted with UPI. In general, the basicity of Arg at neutral or weakly basic pH is not good compared to His, resulting in Arg134‐mediated base catalysis for UPI deprotonation being challenging. Although Arg134 can function as a base, the distance between Arg134 and UPI is not sufficiently close for effective base catalysis. Notably, the distance between His200 and UPI was farther in the *Bs* SirB L200H variant. Thus, His200 could not function as a base catalyst. The position of the ε‐NH moiety of Arg134 in the *Bs* SirB L200H variant was similar to that of the WT; however, this moiety was strongly anchored via polar interaction with the adjacent His200 at a distance of 3.5 Å. This fixation of Arg134 negatively affects the flexible motion of Arg134, possibly resulting in low efficiency of deprotonation of UPI by Arg134 when it can function as a base catalyst.

## Discussion

The *Bs* SirB R134H variant showed a moderately enhanced Ni‐chelatase activity, which is usually lower than Co‐chelatase activity in CfbA. This result indicated that a single His, in the case of His134 of *Bs* SirB, is sufficient for enhancing activity, which was not clarified in the previous proposed mechanism of CfbA, possibly using two His residues as base catalysts. In other words, two His would not be essential for base catalysis toward the tetrapyrrole substrate in Ni‐chelatase function on class II chelatases.

Interestingly, the *Bs* SirB R134H/L200H variant is proposed to be more similar to CfbA from the viewpoint of the His residues; however, this variant exhibited lower activity than the *Bs* SirB R134H variant owing to its partial structural denaturation, as well as slightly less Ni‐binding compared with SirB WT and the other variants. One idea to explain the lower activity is that the four His arrangements in one monomeric class II chelatase may be unfavorable for its protein stability; although CfbA has four His residues in its active site. However, it is still elusive why the four His arrangements are structurally unfavored for SirB but acceptable for CfbA, and the molecular mechanism underlying the partial denaturation of *Bs* SirB R134H/L200H remains unclarified. The modeling of His134 and His200 on *Bs* SirB may not indicate a steric crash, suggesting that the denaturation of *Bs* SirB R134H/L200H may be caused by other reasons, such as partial protein misfolding. Thus, it should be noted that a posed structure shown in the docking simulation could explain only a possible close distance between His134 and UPI, but not other key factors (e.g. degree of protein structural denaturation) related to the activities.

It is also important to consider why *Af* CfbA (or *Af* CbiX^S^) still has higher Ni‐chelatase activity than the SirB R134H variant. We actually expected the R134H/L200H variant could have the highest activity, if its structure had not been denatured. One hypothesis for the higher activity of *Af* CfbA is that two His can deliver protons via His75 of CfbA, when His9 in CfbA deprotonates in adjacent to NH moieties of the tetrapyrrole (Figs. 2 and 5). In other words, if SirB R134H/L200H was structurally stable, His134/His200 may have a function in a similar way to two His out of four His in *Af* CfbA. Thus, maintaining the class II chelatase architecture is an important factor for Ni‐chelatase activity, if the importance of proton‐delivery via two His (His9 and His75 in the case of *Mj* CfbA) is hypothesized.

Interestingly, no studies have revealed a naturally occurring monomeric class II chelatase having four His residues at the active site. This may be understood based on the hypothetical structural basis for the denaturation of a four His‐containing monomeric class II chelatase, as observed in the SirB R134H/L200H variant. The limitation on the number and positions of His residues is important for considering the structural diversity and evolution of class II chelatases. It raises questions about why and how monomeric class II chelatases, which are considered as descendant types, could evolve from the ancestral CfbA via changes in His residues at the active sites through mutations.

## Conflict of interest

The authors declare no conflicts of interest.

### Peer review

The peer review history for this article is available at https://www.webofscience.com/api/gateway/wos/peer‐review/10.1002/2211‐5463.13849.

## Author contributions

TF conceived and designed the project. YO and TF performed mutagenesis, protein expression and purification, and docking simulation. TF performed CD spectroscopy. YO performed activity assay with support from SO. TF wrote the article.

## Supporting information


**Fig. S1.** The overall structure and coordination geometry of Ni^2+^‐bound CfbA.
**Fig. S2.** The overall structure and coordination geometry of Co^2+^‐bound SirB.
**Fig. S3.** SDS‐PAGE of SirB WT, R134H, L200H and R134H/L200H variants.
**Fig. S4.** Gel filtration analysis for determination of molecular weight of SirB WT and variants.
**Fig. S5.** UV–visible spectra in the mixture of UPI and Ni^2+^ without SirB and only the presence of Ni^2+^.
**Fig. S6.** Plots for time‐course changes in the difference in the absorbance at 552 nm in the Ni‐UPI formation by *Bs* SirB R134H variant.
**Fig. S7.** CD spectroscopy for analyzing the binding properties of Ni^2+^ to SirB WT and its variants.
**Table S1.** List of mutagenic primers used in this study.

## Data Availability

Data available within the article and/or the supplementary material.

## References

[1] Bryant DA , Hunter CN and Warren MJ (2020) Biosynthesis of the modified tetrapyrroles‐the pigments of life. J Biol Chem 295, 6888–6925. 32241908 10.1074/jbc.REV120.006194 PMC7242693

[2] Brindley AA , Raux E , Leech HK , Schubert HL and Warren MJ (2003) A story of chelatase evolution: identification and characterization of a small 13‐15‐kDa “ancestral” cobaltochelatase (CbiX^S^) in the archaea. J Biol Chem 278, 2238–22395. 10.1074/jbc.M302468200 12686546

[3] Romão CV , Ladakis D , Lobo SA , Carrondo MA , Brindley AA , Deery E , Matias PM , Pickersgill RW , Saraiva LM and Warren MJ (2011) Evolution in a family of chelatases facilitated by the introduction of active site asymmetry and protein oligomerization. Proc Natl Acad Sci USA 108, 97–102. 21173279 10.1073/pnas.1014298108 PMC3017170

[4] Fujishiro T (2022) Sirohydrochlorin nickelochelatase CfbA. Encyclopaedia Inorg Bioinorg Chem ebic2815.

[5] Dailey HA , Dailey TA , Gerdes S , Jahn D , Jahn M , O'Brian MR and Warren MJ (2017) Prokaryotic heme biosynthesis: multiple pathways to a common essential product. Microbiol Mol Biol Rev 81, e00048‐16. 28123057 10.1128/MMBR.00048-16 PMC5312243

[6] Crouse BR , Sellers VM , Finnegan MG , Dailey HA and Johnson MK (1996) Site‐directed mutagenesis and spectroscopic characterization of human ferrochelatase: identification of residues coordinating the [2Fe‐2S] cluster. Biochemistry 35, 16222–16229. 8973195 10.1021/bi9620114

[7] Sellers VM , Wu CK , Dailey TA and Dailey HA (2001) Human ferrochelatase: characterization of substrate‐iron binding and proton‐abstracting residues. Biochemistry 40, 9821–9827. 11502175 10.1021/bi010012c

[8] Wu CK , Dailey HA , Rose JP , Burden A , Sellers VM and Wang BC (2001) The 2.0 Å structure of human ferrochelatase, the terminal enzyme of heme biosynthesis. Nat Struct Biol 8, 156–160. 11175906 10.1038/84152

[9] Al‐Karadaghi S , Franco R , Hansson M , Shelnutt JA , Isaya G and Ferreira GC (2006) Chelatases: distort to select? Trends Biochem Sci 31, 135–142. 16469498 10.1016/j.tibs.2006.01.001 PMC2997100

[10] Dailey HA , Wu CK , Horanyi P , Medlock AE , Najahi‐Missaoui W , Burden AE , Dailey TA and Rose J (2007) Altered orientation of active site residues in variants of human ferrochelatase. Evidence for a hydrogen bond network involved in catalysis. Biochemistry 46, 7973–7979. 17567154 10.1021/bi700151f PMC2424199

[11] Medlock A , Swartz L , Dailey TA , Dailey HA and Lanzilotta WN (2007) Substrate interactions with human ferrochelatase. Proc Natl Acad Sci USA 104, 1789–1793. 17261801 10.1073/pnas.0606144104 PMC1794275

[12] Medlock AE , Dailey TA , Ross TA , Dailey HA and Lanzilotta WN (2007) A pi‐helix switch selective for porphyrin deprotonation and product release in human ferrochelatase. J Mol Biol 373, 1006–1016. 17884090 10.1016/j.jmb.2007.08.040 PMC2083577

[13] Medlock AE , Carter M , Dailey TA , Dailey HA and Lanzilotta WN (2009) Product release rather than chelation determines metal specificity for ferrochelatase. J Mol Biol 393, 308–319. 19703464 10.1016/j.jmb.2009.08.042 PMC2771925

[14] Gupta V , Liu S , Ando H , Ishii R , Tateno S , Kaneko Y , Yugami M , Sakamoto S , Yamaguchi Y , Nureki O *et al*. (2013) Salicylic acid induces mitochondrial injury by inhibiting ferrochelatase heme biosynthesis activity. Mol Pharmacol 84, 824–833. 24043703 10.1124/mol.113.087940

[15] Leech HK , Raux‐Deery E , Heathcote P and Warren MJ (2002) Production of cobalamin and sirohaem in *Bacillus megaterium*: an investigation into the role of the branchpoint chelatases sirohydrochlorin ferrochelatase (SirB) and sirohydrochlorin cobalt chelatase (CbiX). Biochem Soc Trans 30, 610–613. 12196147 10.1042/bst0300610

[16] Raux E , Leech HK , Beck R , Schubert HL , Santander PJ , Roessner CA , Scott AI , Martens JH , Jahn D , Thermes C *et al*. (2003) Identification and functional analysis of enzymes required for precorrin‐2 dehydrogenation and metal ion insertion in the biosynthesis of sirohaem and cobalamin in *Bacillus megaterium* . Biochem J 370, 505–516. 12408752 10.1042/BJ20021443 PMC1223173

[17] Raux‐Deery E , Leech HK , Nakrieko KA , McLean KJ , Munro AW , Meathcote P , Rigby SE , Smith AG and Warren MJ (2005) Identification and characterization of the terminal enzyme of siroheme biosynthesis from *Arabidopsis thaliana*: a plastid‐located sirohydrochlorin ferrochelatase containing a 2Fe‐2S center. J Biol Chem 280, 4713–4721. 15545265 10.1074/jbc.M411360200

[18] Fujishiro T , Shimada Y , Nakamura R and Ooi M (2019) Structure of sirohydrochlorin ferrochelatase SirB: the last of the structures of the class II chelatase family. Dalton Trans 48, 6083–6090. 30778451 10.1039/c8dt04727h

[19] Leech HK , Raux E , McLean KJ , Munro AW , Robinson NJ , Borrelly GP , Malten M , Jahn D , Rigby SE , Heathcote P *et al*. (2003) Characterization of the cobaltochelatase CbiX^L^: evidence for a 4Fe‐4S center housed within an MXCXXC motif. J Biol Chem 278, 41900–41907. 12917443 10.1074/jbc.M306112200

[20] Moore SJ , Lawrence AD , Biedendieck R , Deery E , Frank S , Howard MJ , Rigby SE and Warren MJ (2013) Elucidation of the anaerobic pathway for the corrin component of cobalamin (vitamin B12). Proc Natl Acad Sci USA 110, 14906–14911. 23922391 10.1073/pnas.1308098110 PMC3773766

[21] Lobo SA , Brindley AA , Romão CV , Leech HK , Warren MJ and Saraiva LM (2008) Two distinct roles for two functional cobaltochelatases (CbiK) in *Desulfovibrio vulgaris* Hildenborough. Biochemistry 47, 5851–5857. 18457416 10.1021/bi800342c

[22] Lobo SA , Videira MA , Pacheco I , Wass MN , Warren MJ , Teixeira M , Matias PM , Romão CV and Saraiva LM (2017) *Desulfovibrio vulgaris* CbiK^P^ cobaltochelatase: evolution of a haem binding protein orchestrated by the incorporation of two histidine residues. Environ Microbiol 19, 106–118. 27486032 10.1111/1462-2920.13479

[23] Zheng KY , Ngo PD , Owens VL , Yang XP and Mansoorabadi SO (2016) The biosynthetic pathway of coenzyme F430 in methanogenic and methanotrophic archaea. Science 354, 339–342. 27846569 10.1126/science.aag2947

[24] Moore SJ , Sowa ST , Schuchardt C , Deery E , Lawrence AD , Ramos JV , Billig S , Birkemeyer C , Chivers PT , Howard MJ *et al*. (2017) Elucidation of the biosynthesis of the methane catalyst coenzyme F_430_ . Nature 543, 78–82. 28225763 10.1038/nature21427 PMC5337119

[25] Pisarchik A , Petri R and Schmidt‐Dannert C (2007) Probing the structural plasticity of an archaeal primordial cobaltochelatase CbiX^S^ . Protein Eng Des Sel 20, 257–265. 17584754 10.1093/protein/gzm018

[26] Fujishiro T and Ogawa S (2021) The nickel‐sirohydrochlorin formation mechanism of the ancestral class II chelatase CfbA in coenzyme F430 biosynthesis. Chem Sci 12, 2172–2180. 34163982 10.1039/d0sc05439a PMC8179277

[27] Schuelke‐Sanchez AE , Stone AA and Liptak MD (2020) CfbA promotes insertion of cobalt and nickel into ruffled tetrapyrroles *in vitro* . Dalton Trans 49, 1065–1076. 31868194 10.1039/c9dt03601f

[28] Trott O and Olson AJ (2010) AutoDock Vina: improving the speed and accuracy of docking with a new scoring function, efficient optimization, and multithreading. J Comput Chem 31, 455–461. 19499576 10.1002/jcc.21334 PMC3041641

[29] Schüttelkopf AW and van Aalten DM (2004) PRODRG: a tool for high‐throughput crystallography of protein‐ligand complexes. Acta Crystallogr Sect D 60, 1355–1363. 15272157 10.1107/S0907444904011679

[30] Yin J , Xu LX , Cherney MM , Raux‐Deery E , Bindley AA , Savchenko A , Walker JR , Cuff ME , Warren MJ and James MN (2006) Crystal structure of the vitamin B12 biosynthetic cobaltochelatase, CbiX^S^, from *Archaeoglobus fulgidus* . J Struct Funct Genom 7, 37–50. 10.1007/s10969-006-9008-x 16835730

[31] Aflano M , Pérard J , Carpentier P , Basset C , Zambelli B , Timm J , Crouzy S , Ciurli S and Cavazza C (2019) The carbon monoxide dehydrogenase accessaory protein CooJ is a histidine‐rich multidomain dimer containing an unexpected Ni(II)‐binding site. J Biol Chem 294, 7601–7614. 30858174 10.1074/jbc.RA119.008011 PMC6514639

