# Supplementary material for: Nickel‐chelatase activity of SirB variants mimicking the His arrangement in the naturally occurring nickel‐chelatase CfbA
Source: FEBS Open Bio. 2024 Jun 24;14(8):1291–302. doi: 10.1002/2211-5463.13849 (PMC11301274; doi:10.1002/2211-5463.13849)
Supplement: Supplementary file 1 — Fig. S1. The overall structure and coordination geometry of Ni2+‐bound CfbA. Fig. S2. The overall structure and coordination geometry of Co2+‐bound SirB. Fig. S3. SDS‐PAGE of SirB WT, R134H, L200H and R134H/L200H variants. Fig. S4. Gel filtration analysis for determination of molecular weight of SirB WT and variants. Fig. S5. UV–visible spectra in the mixture of UPI and Ni2+ without SirB and only the presence of Ni2+. Fig. S6. Plots for time‐course changes in the difference in the absorbance at 552 nm in the Ni‐UPI formation by Bs SirB R134H variant. Fig. S7. CD spectroscopy for analyzing the binding properties of Ni2+ to SirB WT and its variants. Table S1. List of mutagenic primers used in this study. [file FEB4-14-1291-s001.pdf]

Supporting information

Nickel-chelatase activity of SirB variants mimicking the His arrangement in the naturally occurring nickel-chelatase CfbA

Yuuma Oyamada, Shoko Ogawa and Takashi Fujishiro*

*Corresponding author: Takashi Fujishiro
Department of Biochemistry and Molecular Biology
Graduate School of Science and Engineering, Saitama University,
Shimo-okubo 255, Sakura-ku, Saitama 38-8570, Japan
E-mail: tfujishiro@mail.saitama-u.ac.jp
Tel: +81-48-858-9293

Table S1. List of mutagenic primers used in this study

Primer	Sequence (5′→3′)
Inv-BsSirB-R134H-for	5′-CACGGAAGTTCAGATCCAGACGTCAAAAGAGACG-3′
Inv-BsSirB-R134H-rev	5′-TCCGATGAGCACCACTCTGGCATTTTCATAC-3′
Inv-BsSirB-L200H-for	5′-CACATGAATGAAATCGAACGAGAGGTTCAGAAATTAAAA-3′
Inv-BsSirB-L200H-rev	5′-CATACCCGTAAAAAGCAAATATGGAACGATAAAGGTTG-3′

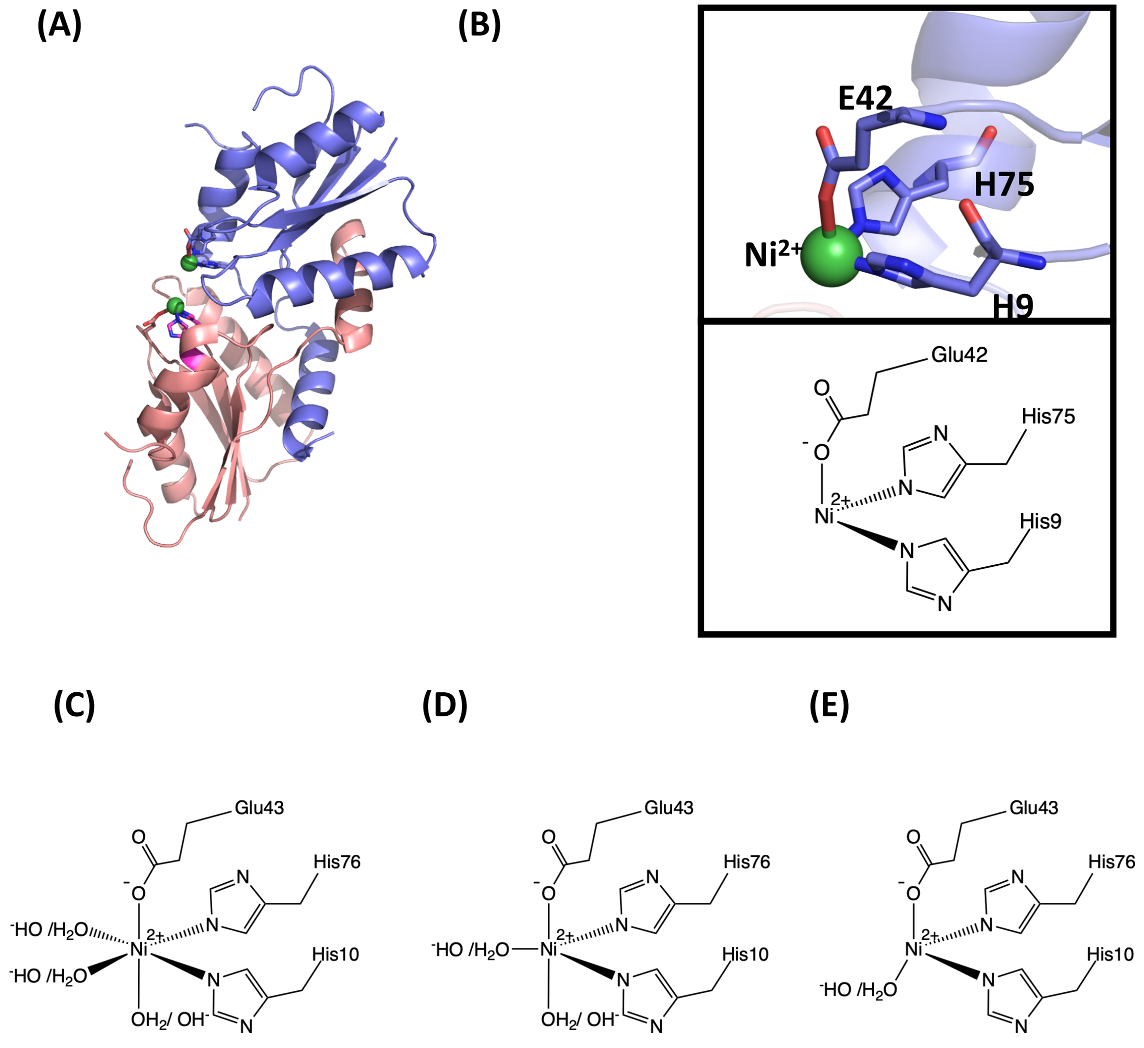

Fig. S1. The overall structure and coordination geometry of Ni^{2+}-bound CfbA. (A) The overall structure (PDB ID: 6M27). (B) The coordination structure and chemical structure of the Ni-binding site. It is noted that other possible ligands such as H_2O/OH^- are not visible due to the low resolution of the Ni^{2+}-bound CfbA structure, although Ni-coordination is normally 4-, 5-, or 6-coordination. (C, D, E) Proposed chemical structures of (C) 6-coordination, (D) 5-coordination, and (E) 4-coordination of Ni-binding site.

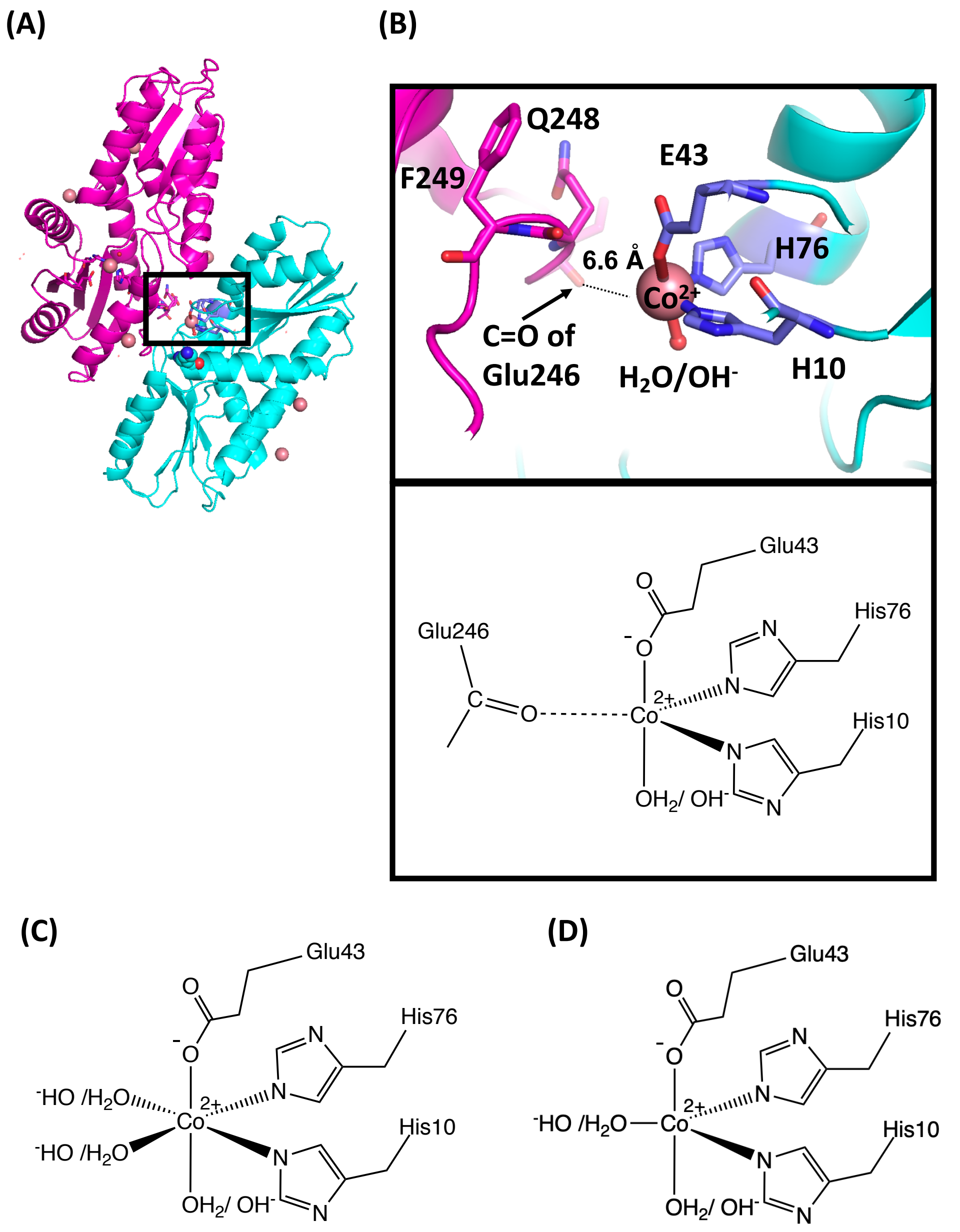

Fig. S2. The overall structure and coordination geometry of Co^{2+}-bound SirB. (A) The overall structure (PDB ID: 5ZT7). (B) The coordination structure and chemical structure of the Ni-binding site. It is noted that only one H_2O/OH^- molecule is visible, but the others not, due to the low resolution. Also, the crystal packing of this SirB structure results in the C=O moiety of Glu246 of one SirB molecule is located near the Co^{2+} of the other SirB molecule. (C, D) Proposed chemical structures of (C) 6-coordination, (D) 5-coordination, based on the structure of (B).

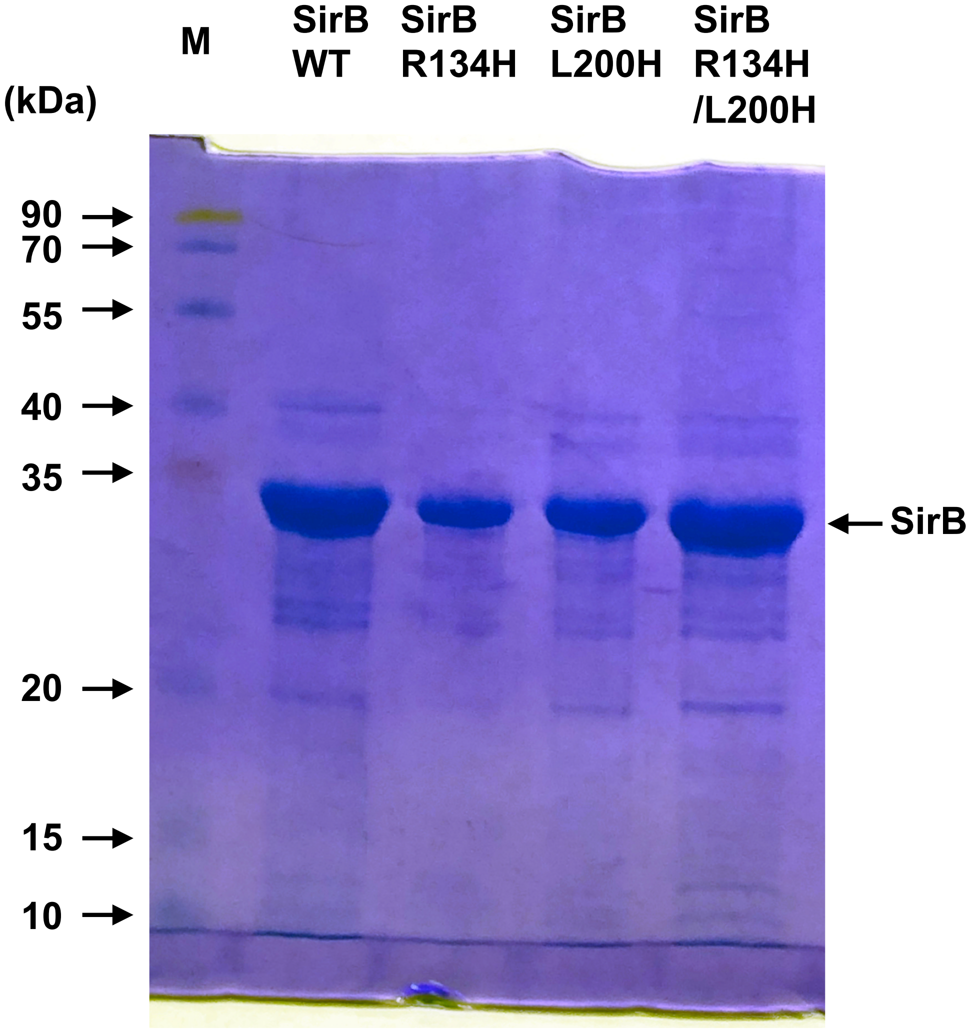

Fig. S3. SDS-PAGE of SirB WT, R134H, L200H and R134H/L200H variants.

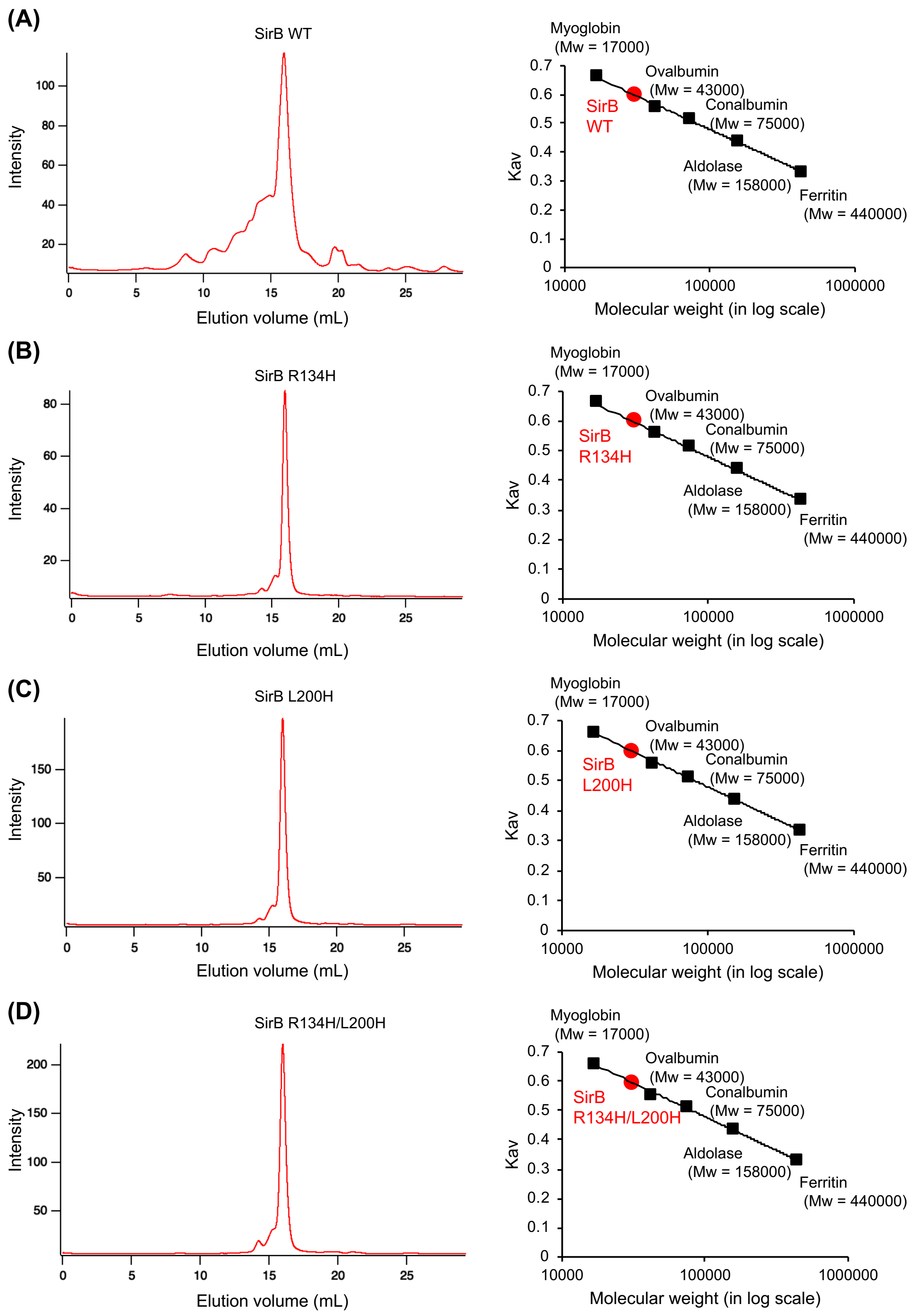

Fig. S4. Gel filtration analysis for determination of molecular weight of SirB WT and variants. (A) SirB WT. (B) SirB R134H. (C) SirB L200H. (D) SirB R134H/L200H.

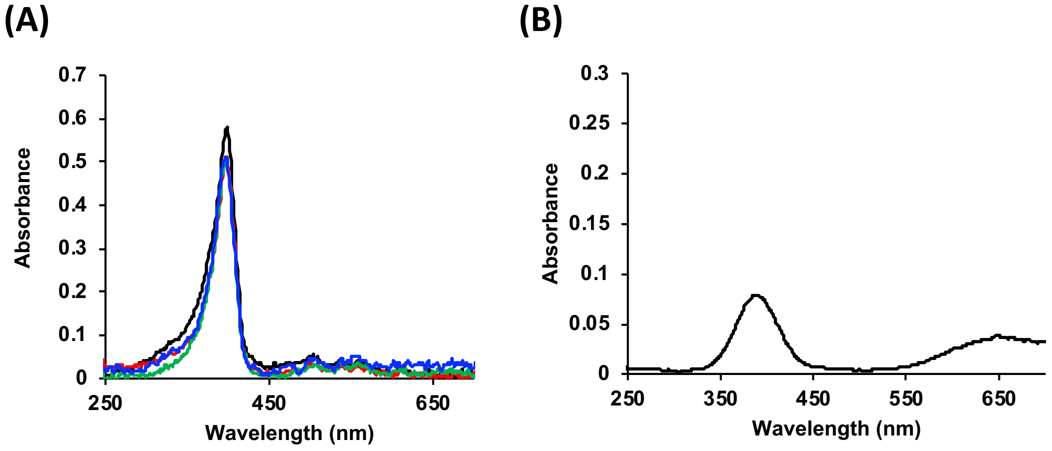

Fig. S5. (A) Time-course monitoring of UV-visible spectra in the mixture of UPI and Ni^{2+}. The Sore and Q-bands were not changed in the absence of SirB. Reaction time:0 h (black line), 24 h (red line), 48 h (green line) and 72 h (blue line). The reaction conditions were as follows: 5 μM of UPI, 200 μM of $NiCl_2$ in 50 mM Tris-HCl buffer, pH 8.0, containing 150 mM NaCl under dark at room temperature. (B) UV-visible spectrum of 10 mM $NiCl_2$ in 50 mM Tris-HCl buffer, pH 7.8. The peak derived from the ligand-to-metal charge transfer (LMCT) was found at 394 nm. The spectrum was recorded in a 1 cm-cell path quartz cuvette containing an 1 mL volume of 10 mM $NiCl_2$ solution at room temperature.

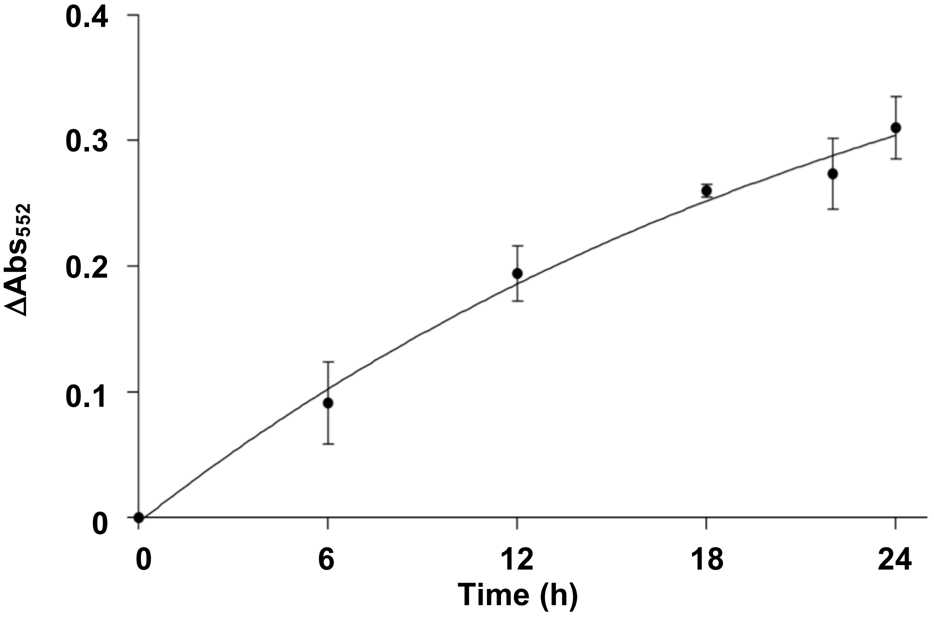

Fig. S6. Plots for time-course changes in the difference in the absorbance at 552 nm in the Ni-UPI formation by *Bs* SirB R134H variant. Experiments for each plot were performed three times. Error bars for the plots indicate the standard deviations. Curve-fitting was based on the pseudo-first order kinetic equation.

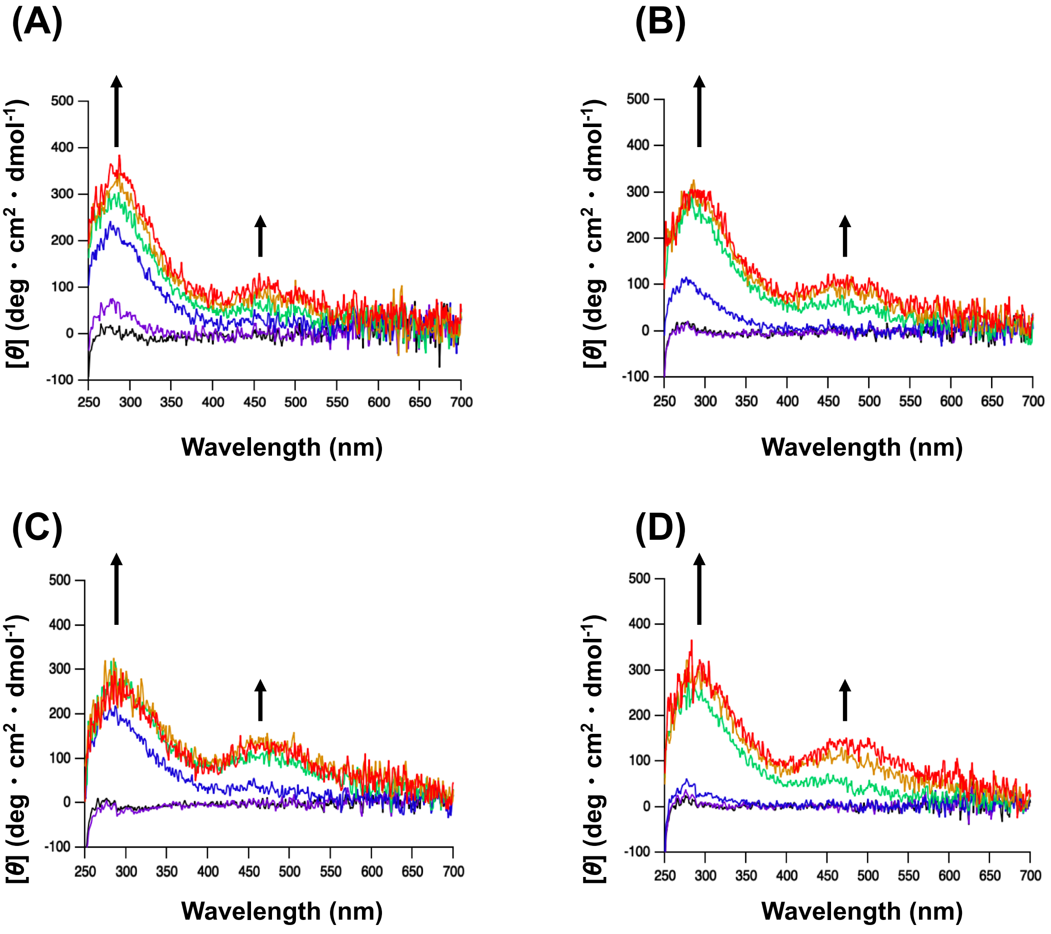

Fig. S7. CD spectroscopy for analyzing the binding properties of Ni^{2+} to SirB WT and its variants. (A) SirB WT. (B) SirB R134H. (C) SirB L200H. (D) SirB R134H/L200H. Arrows indicated the CD spectral changes upon the addition of $NiCl_2$ to SirB enzymes. Each of the spectra in different colors indicated the difference concentrations of $NiCl_2$ added to SirB samples. Concentrations of $NiCl_2$ were as follows: 0 μM (black line), 10 μM (purple line), 20 μM (blue line), 30 μM (green line), 40 μM (orange line), and 60 μM (red line).
